# Auditory Neuropathy Spectrum Disorder due to Two Novel Compound Heterozygous *OTOF* Mutations in Two Chinese Families

**DOI:** 10.1155/2019/9765276

**Published:** 2019-11-18

**Authors:** Yue Qiu, Sen Chen, Le Xie, Kai Xu, Yi Lin, Xue Bai, Hui-Min Zhang, Xiao-Zhou Liu, Yuan Jin, Yu Sun, Wei-Jia Kong

**Affiliations:** ^1^Department of Otorhinolaryngology, Union Hospital, Tongji Medical College, Huazhong University of Science and Technology, Wuhan 430022, China; ^2^Institute of Otorhinolaryngology, Tongji Medical College, Huazhong University of Science and Technology, 430022 Wuhan, China

## Abstract

Auditory neuropathy spectrum disorder (ANSD), also called auditory neuropathy (AN), is a unique type of prelingual hearing impairment. Up to 10% of deaf infants and children are affected by this disease. Mutation of the *OTOF* gene which encodes otoferlin is the common cause of congenital nonsyndromic ANSD. To date, over 110 mutations have been identified in the *OTOF* gene according to the Human Gene Mutation Database (HGMD). Here, next-generation sequencing (NGS) revealed that the compound heterozygous mutations c.4748G>A/c.2523+1G>T and c.5248G>C/c.5098G>C of the *OTOF* gene were present in two Chinese ANSD patients. Each patient had a known pathogenic mutation (c.4748G>A or c.5098G>C) and a novel mutation (c.2523+1G>T or c.5248G>C). Comparative amino acid sequence analysis across different species revealed that the residues at these novel mutation sites are evolutionarily highly conservative. This indicated that the novel mutations were possible causes of the disorder in the patients. Our findings extend the *OTOF* mutation spectrum and further confirm the role of the *OTOF* gene in ANSD.

## 1. Introduction

Auditory neuropathy spectrum disorder (ANSD), also called auditory neuropathy (AN), is a unique type of prelingual hearing impairment. The prevalence of ANSD varies from 0.23% to 24% in different countries [1–4], and up to 10% of deaf infants and children are affected by this disease [2]. ANSD can be inherited in both syndromic and nonsyndromic conditions. There are a vast number of etiological factors of the disease, among which genetic factors account for approximately 40% of cases [1, 3]. The most frequent genetic causes of nonsyndromic ANSD are mutations of the *OTOF*, *DFNB59*, and *DIAPH3* genes [2]. Among nonsyndromic ANSD patients, *OTOF* mutations account for 18% in Chinese [5], 57% in Japanese [6], 20% in Korean [7], 27% in Brazilian [8], and 87% in Spanish [9]. Further, according to Zhang et al., more than 41% of congenital ANSD cases in China are caused mainly by *OTOF* mutations [10]. To date, over 110 mutations in the *OTOF* gene have been recorded in the Human Gene Mutation Database (HGMD) (http://www.hgmd.cf.ac.uk/ac/index.php).

The *OTOF* gene, first identified in 1999 [11], is located on chromosome 2p23.1. It consists of 48 exons and encodes otoferlin [12]. Otoferlin is mainly expressed in the inner hair cells (IHCs) of adult mammalian cochlea and is located in the basolateral region, participating in afferent synaptic contacts [13]. It is a member of the ferlin protein family and has two classes of isoforms. The long isoforms contain six C2 domains (C2A-C2F) and a C-terminal transmembrane domain (TMD), and the short isoforms contain only the last three C2 domains (C2D-C2F) and a TMD. The C2 domains are highly conserved, and the last four domains have five conserved aspartyl residues predicted to bind Ca^2+12^. This suggests that otoferlin is involved in docking of the synaptic vesicles to the plasma membrane and mediating their fusion and exocytosis in a Ca^2+^-dependent manner in rodents [13–16].

As the mutation spectrum of the *OTOF* gene shows great discrepancies between different countries and ethnic populations, including high frequencies of the mutation p.E1700Q in Chinese from Taiwan [5], p.R1939Q in Japanese [6, 17], p.Val1778Phe in Ashkenazi Jewish [18], Q829X in Spanish [19], c.2905-2923delinsCTCCGAG-CGCA in Argentinean [9], and p.R1792H in Saudi Arabian [20], understanding of the molecular mechanism of ANSD needs to be improved. Here, we report two novel compound heterozygous *OTOF* mutations in different Chinese families, which provide more information about the etiology of ANSD at the molecular level.

## 2. Materials and Methods

### 2.1. Family Description

**Figure 1:**
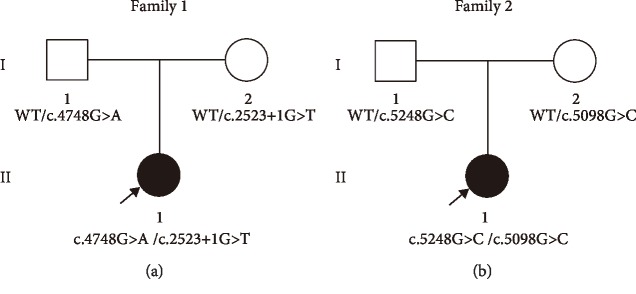
Pedigree of affected Family 1 (a) and Family 2 (b) with ANSD. Sequencing analysis showed that Family member 1-II-1 had the compound heterozygous mutation, c.4748G>A/c.2523+1G>T, and Family member 2-II-1 had the compound heterozygous mutation, c.5248G>C/c.5098G>C. Their parents were heterozygous carriers. Probands are denoted in black (indicated by arrows). WT: wild-type.

Family 1 and Family 2 are two unrelated Chinese families each containing one member with autosomal recessive nonsyndromic hearing loss (ARNHL) (Figure 1(a) and Figure 1(b)). Family member 1-II-1, a child aged one year and 3 months, failed to pass hearing screening and was diagnosed with ANSD at 4 months of age. Family member 2-II-1, a 7-month-old child, passed the neonatal hearing screen (NHS), but was subsequently found to have hearing loss and diagnosed with ANSD. The parents of both probands had no history of hearing impairment.

### 2.2. Clinical Examination

Both probands underwent audiological examination including otoscopic examination, behavioral observation audiometry (BOA), auditory brainstem response (ABR), distortion product otoacoustic emission (DPOAE), auditory immittance, and auditory steady-state evoked response (ASSR). Computed tomography (CT) scan of the temporal bone and magnetic resonance imaging (MRI) were also performed. According to the international consensus (ICON) on audiological assessment of hearing loss in children 2018, absent or severely abnormal ABR and present otoacoustic emission (OAE) and/or cochlear microphonic (CM) testing are recommended to diagnose infants and young children with ANSD. Informed consent was obtained from both patients for inclusion in the study.

### 2.3. Mutation Detection and Analysis

Deafness panel sequencing/NGS+Sanger sequencing were performed by MyGenostics Inc. (Beijing, China). For NGS+Sanger sequencing, peripheral venous blood samples were obtained from all the family members. Genomic DNA was extracted from the blood samples using a QIAamp DSP DNA Blood Mini Kit (61104, Qiagen Inc., Valencia, CA, USA) and fragmented to a size of approximately 350–400 base pairs using a Bioruptor sonicator (Diagenode, Liège, Belgium). Then, end-repair and Illumina adaptor ligation were performed for library construction according to Illumina protocols. After completing polymerase chain reaction (PCR), targeted DNA fragments were captured by biotinylated single-strand DNA capture probes (MyGenostics, Beijing, China) and sequenced on an Illumina HiSeq2000 Analyzer for automated cycles per read. The probes were designed to tile along the exon regions and exon-intron boundaries of the 162 known deafness-related nuclear genes as well as deafness-related mitochondrial and microRNA regions. Initial data were generated on TrimGalore software (version 0.4.3; Babraham Bioinformatics, The Babraham Institute, Cambridge, UK). Illumina sequencing adapters and low quality reads (<80 bp) were filtered using cutadapt (http://cutadapt.readthedocs.io/en/stable/). After quality control, the clean reads were aligned to the Genome Reference Consortium Human genome build 37 (GRCh37)/Human genome build 19 (hg19) using Burrows-Wheeler Aligner (http://bio-bwa.sourceforge.net/). Duplicated reads were removed using picard tools, and mapping reads were used for variation detection. The variants of single nucleotide polymorphisms (SNPs) and inserts and deletions (InDels) were detected by GATK Haplotype Caller; then, GATK Variant Filtration was used to filter variants. After the two steps above, variants were further annotated by ANNOVAR (http://annovar.openbioinformatics.org/en/latest/) and compared with multiple databases including 1000 Genomes (http://phase3browser.1000genomes.org/index.html), NHLBI Exome Sequencing Project 6500 (ESP6500) (http://evs.gs.washington.edu/EVS), Database of Single Nucleotide Polymorphisms (dbSNP) (http://www.ncbi.nlm.nih.gov/projects/SNP/), HGMD (http://www.hgmd.cf.ac.uk/ac/index.php), and Exome Aggregation Consortium (ExAC) (http://exac.broadinstitute.org/). Filtered candidate variants were confirmed by Sanger sequencing. The coding exons that contained the detected mutations were amplified with ExTaq DNA polymerase (Takara, Dalian, China). Purified PCR samples were sequenced on an ABI 3730 Genetic Analyzer (Applied Biosystems, Foster City, CA, USA). Sequence traces were analyzed using the Mutation Surveyor (Softgenetics LLC, State College, PA, USA). The mutations of family members were confirmed by the same procedure. This study used the method we used in our previous research, and the method description partly reproduces the wording [21]. The complete nucleotide sequence of the *OTOF* gene in each species is available in the National Center for Biotechnology Information (NCBI) GenBank database (https://www.ncbi.nlm.nih.gov/nuccore/).

### 2.4. Structural Model Building and Analysis

Homologous tertiary structural models of human wild-type and mutational otoferlin were built up using SWISS-MODEL (https://swissmodel.expasy.org/). The complete amino acid sequence of *OTOF* in each species is available in the NCBI GenBank database (https://www.ncbi.nlm.nih.gov/nuccore/).

## 3. Results

### 3.1. Clinical Data

**Figure 2:**
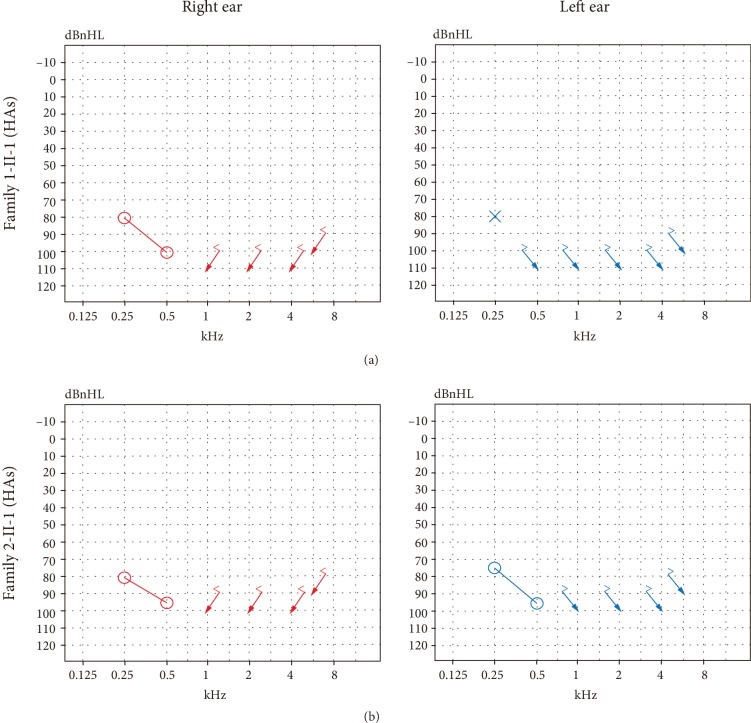
Results of sound field threshold tests after fitting of hearing aids (HAs). (a) Sound field threshold of proband 1-II-1 after hearing aids. (b) Sound field threshold of proband 2-II-1 after fitting of hearing aids.

For proband 1-II-1, BOA showed poor hearing and tympanograms were type A (left ear) and type As (right ear). The wave V thresholds of ABR were 90 dB nHL (left ear) and 95 dB nHL (right ear) of proband 1-II-1 while there were no waves at either ear of proband 2-II-1. For both probands, DPOAEs were present in both ears. However, both of them showed poor hearing as before, even after hearing aids were fitted (Figure 2). ASSR showed the thresholds were 85 dB nHL at 500 Hz, 80 dB nHL at 1 kHz (left ear), 65 dB nHL at 500 Hz, and 60 dB nHL at 1 kHz (right ear) of proband 1-II-1; 70 dB nHL at 500 Hz, 75 dB nHL at 1 kHz (left ear), 85 dB nHL at 500 Hz, and 100 dB nHL at 1 kHz (right ear) of proband 2-II-1 (audiological examination results are summarized in Table 1). No abnormalities were found by CT or MRI in the two affected children.

### 3.2. Mutation Identification and Analysis

**Figure 3:**
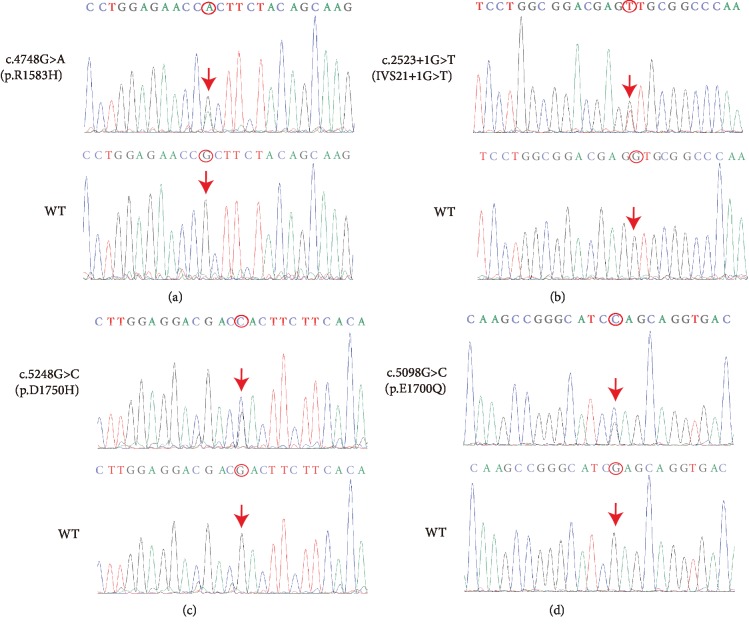
Wild-type (below) and mutated (above) *OTOF* sequences. c.4748G>A (p.R1583H) (a), c.2523+1G>T (IVS21+1G>T) (b), c.5248G>C (p.D1750H) (c), and c.5098G>C (p.E1700Q) (d). Red arrows and circles: sites of nucleotide changes. WT: wild-type.

The 162 known deafness-related nuclear genes as well as deafness-related mitochondrial and microRNA regions were sequenced using NGS. Mutations in *DFNB59*, *DIAPH3*, *GJB2*, and mitochondrial 12S rRNA genes which are reported to be causes of nonsyndromic ANSD [2] were excluded. After aligning to the human reference genome (GRCh37/hg19), two novel compound heterozygous mutations c.4748G>A/c.2523+1G>T and c.5248G>C/c.5098G>C were found in proband 1-II-1 and proband 2-II-1 (Figure 1), respectively. The variants detected in the two families are shown in Table 2. Among them, two mutations were novel: c.2523+1G>T(IVS21+1G>T) and c.5248G>C (p.D1750H), while the other two mutations were previously identified: c.4748G>A (p.R1583H) and c.5098G>C (p.E1700Q) [5, 17, 22]. The c.4748G>A and c.5098G>C mutations occurred in exon 38 and exon 40 with mutations of No. 4748 nucleotide from guanine to adenine (Figure 3(a)) and No. 5098 nucleotide from guanine to cytosine (Figure 3(d)), respectively. The c.2523+1G>T was a splice mutation of No. 2523+1 nucleotide from guanine to thymine (Figure 3(b)), and the c.5248G>C in exon 42 was a missense mutation of No. 5248 nucleotide from guanine to cytosine (Figure 3(c)). None of these mutations were polymorphic sites. The variants (IVS21+1G>T and p.D1750H) were not listed in 1000 Genomes, ESP6500, and ExAC, and have not been reported before. The prevalence of variant p.R1583H was 0.000077 in EPS6500, 0.0000082 in ExAC. However, it was not listed in 1000 Genomes. The prevalence of variant p.E1700Q was 0.0015974 in 1000 Genomes, 0.0005 in ExAC, but was not listed in EPS6500. The sequencing results showed that their parents were all heterozygous carriers (Figures 1(a) and 1(b)). The American College of Medical Genetics and Genomics (ACMG) guidelines recommend the use of specific standard terminology to describe variants identified in genes that cause Mendelian disorders, including pathogenic, likely pathogenic, uncertain significance, likely benign, and benign [23]. According to the ACMG criteria, the c.4748G>A, c.2523+1G>T, and c.5098G>C variants were likely pathogenic, while the c.5248G>C variant had uncertain significance.

### 3.3. Structure Modeling

**Figure 4:**
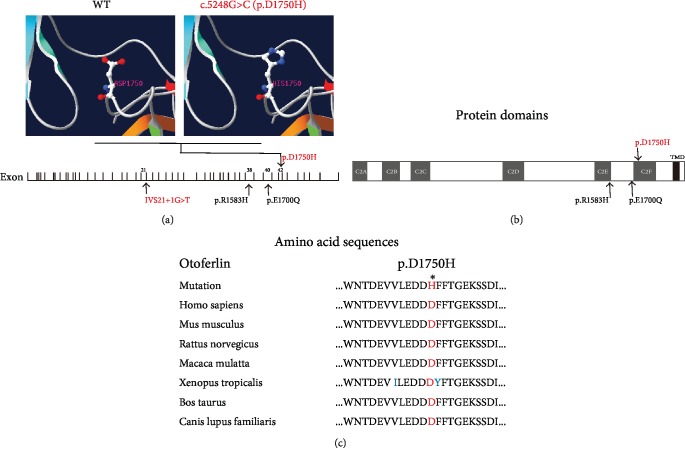
Predicted protein structures and evolutionary conservation of amino acids affected by the missense and splice site mutations. (a) Local predicted protein tertiary structures of human otoferlin with p.D1750H. Left: amino acid side chain of wild-type p.D1750. Right: amino acid side chain of mutation p.H1750. Schematic diagram of 48 exons of human otoferlin (below) is shown with all pathogenic mutations (arrows) of two families. Novel compound heterozygous *OTOF* mutations are indicated in red. Previously reported mutations are indicated in black. (b) Schematic diagram of functional domains of human otoferlin with pathogenic mutations of p.R1583H, p.D1750H, and p.E1700Q (shown by arrows). Six Ca^2+^-binding domains (C2A-C2F) are indicated by gray boxes. The C-terminal transmembrane domain (TMD) is indicated by a black box. Novel compound heterozygous *OTOF* mutations are indicated in red. Previously reported mutations are indicated in black. (c) Evolutionary conservation of No. 1750 amino acid (red). Asterisks: mutation sites. Different residues are shown in blue.

Protein tertiary structures were modeled using SWISS-MODEL (http://www.swissmodel.expasy.org/), which predicted the sequence homology. All of the p.R1583H, p.D1750H, and p.E1700Q protein models covered the target sequence (residues 1492–1896). The basis of the constructed protein was receptor-type protein tyrosine phosphatase S (PDB ID: 1dqv.1.A). Sequence identity between the target and template was 23.86%. The p.R1583H, p.D1750H, and p.E1700Q protein models, respectively, showed substitution of No. 1583 amino acid from arginine to histidine, No. 1750 amino acid from aspartic acid to histidine, and No. 1700 amino acid from glutamic acid to glutamine (Figure 4(a), Supplementary Figures [Supplementary-material supplementary-material-1] and [Supplementary-material supplementary-material-1]). There was no change in hydrogen bonding between the amino acids of p.R1583H (Supplementary [Supplementary-material supplementary-material-1]). The p.E1700Q led to production of hydrogen bonds among No. 1700 glutamine, No. 1701 glutamine, and No. 1702 glycine (Supplementary [Supplementary-material supplementary-material-1]). As the homologous model of IVS21+1G>T had low coverage which did not cover the mutation site, the matched model was not available. The location of each mutation in the *OTOF* gene and the evolutionary conservation of amino acids related to the novel mutation (p.D1750H) are shown in Figure 4.

## 4. Discussion

Two novel compound heterozygous mutations c.4748G>A/c.2523+1G>T and c.5248G>C/c.5098G>C in the *OTOF* gene were identified as possible causes of the ANSD in our patients. In a series of audiological tests, two probands exhibited absent or abnormal ABR and ASSR, while DPOAE of both ears was present (Table 1). According to the diagnostic criteria above, proband 1-II-1 and proband 2-II-1 were diagnosed with ANSD. Next, NGS+Sanger sequencing were used to determine the molecular pathogenic mechanism of these patients. Here, mutations of *DFNB59*, *DIAPH3*, *GJB2*, and mitochondrial 12S rRNA genes were excluded as they are reported to be causes of nonsyndromic ANSD [2]. Four *OTOF* mutations, c.4748G>A (p.R1583H), c.2523+1G>T (IVS21+1G>T), c.5248G>C (p.D1750H), and c.5098G>C (p.E1700Q), were found in these family members. Among them, IVS21+1G>T and p.D1750H were novel while the other two mutations had been previously reported. p.R1583H had been reported to be in a compound heterozygous state with p.Q1883X in Chinese [22] or p.R1939Q in Japanese [17] patients, and had been identified as the pathogenic factor of ANSD. In our study, p.R1583H was found to be heterozygous with IVS21+1G>T in proband 1-II-1. According to the ACMG criteria, p.R1583H and IVS21+1G>T were likely pathogenic variants. The homozygote p.E1700Q has been reported to be a common causative mutation in ANSD patients from Taiwan, China. A heterozygote with p.L912L was also identified as the cause of ANSD in their research [5]. Here, p.E1700Q was compound heterozygous with p.D1750H. According to the ACMG criteria, p.E1700Q was a likely pathogenic variant. p.D1750H was judged as having uncertain significance, but the amino acid at the novel mutation site (p.D1750H) was found to be highly conserved in species through amino acid sequence analysis (Figure 4(c)). Both novel mutations (IVS21+1G>T and p.D1750H) were not polymorphic sites and not listed in 1000 Genomes, ESP6500, or ExAC. We found that their parents were all heterozygous carriers without hearing impairment (Figures 1(a) and 1(b)). These data suggested that IVS21+1G>T and p.D1750H were highly likely to be involved in the pathogenesis of ANSD.

*OTOF* mutations may affect the structure and/or function of otoferlin leading to abnormal synaptic communication and the onset of ANSD. p.R1583H is located in functional domain C2E (Figure 4(b)). It may affect the amino acid side chain due to substitution of arginine to histidine (Supplementary [Supplementary-material supplementary-material-1]), causing functional damage of C2E. p.E1700Q is found in the area between the C2E and C2F domains and protein modeling showed formation of hydrogen bonds among No. 1700 glutamine, No. 1701 glutamine, and No. 1702 glycine (Figure 4(b), Supplementary [Supplementary-material supplementary-material-1]). Additional hydrogen bonds may result in dysfunction of otoferlin. Both of two amino acid sites are highly conserved in different species [5, 17, 22]. The matched homologous model of IVS21+1G>T was not available. However, as shown in Figure 4(a), IVS21+1G>T occurred in the splice site of exon 21/intron 21 which may lead to deleterious splicing. No. 1750 aspartic acid is located in the C2F domain and is highly conserved in Homo sapiens, Mus musculus, Rattus norvegicus, Xenopus tropicalis, Macaca mulatta, Bos taurus, and Canis lupus familiaris (Figure 4(c)). In addition, the aspartic acid residue is predicted to be a Ca^2+^-binding site [12, 13]. Therefore, we speculated that p.D1750H resulted in failure to bind Ca^2+^, thus affecting Ca^2+^-dependent exocytosis and membrane fusion.

## 5. Conclusions

In summary, we successfully identified two novel compound heterozygous *OTOF* mutations, c.4748G>A/c.2523+1G>T and c.5248G>C/c.5098G>C, as the genetic causes of two cases of ANSD using next-generation sequencing+Sanger sequencing. Our findings extend the mutation spectrum and further confirm the key role of the *OTOF* gene in ANSD.

## Figures and Tables

**Table 1:** Results of audiological examination of the two probands.

Subjects	ABR	DPOAE	ASSR	
			Right ear	Left ear
Family 1-II-1	Abnormal	Bil present	Moderate-severe	Severe
Family 2-II-1	Absent	Bil present	Profound	Severe

ABR: auditory brainstem response; DPOAE: distortion product otoacoustic emissions; ASSR: auditory steady-state evoked responses; Bil: bilateral.

**Table 2:** The variants detected in two families.

Gene	Transcript	Chromosome location (GRCh37/hg19)	Nucleotide changes	Amino acid changes
OTOF	NM_194248	chr2:26688591	c.4748G>A	p.R1583H
OTOF	NM_194248	chr2:26700039	c.2523+1G>T	Splicing
OTOF	NM_194248	chr2:26684994	c.5248G>C	p.D1750H
OTOF	NM_194248	chr2:26686837	c.5098G>C	p.E1700Q

## Data Availability

The data used to support the findings of this study are included within the article and the supplementary information file.

## Supplementary Materials

Supplementary Materials See Supplementary Figure 1 in the Supplementary Material. Local predicted protein tertiary structures of human otoferlin with p.R1583H and p.E1700Q. (a) Amino acid side chain of p.R1583H. Left: wild-type p.R1583. Right: mutation p.H1583. (b) Amino acid side chain of p.E1700Q. Left: wild-type p.E1700. Right: mutation p.Q1700. WT: wild-type.
