# Supplementary figures and images for: Auditory Neuropathy Spectrum Disorder due to Two Novel Compound Heterozygous OTOF Mutations in Two Chinese Families
Source: Neural Plast. 2019 Nov 18;2019:9765276. doi: 10.1155/2019/9765276 (PMC6885821; doi:10.1155/2019/9765276)

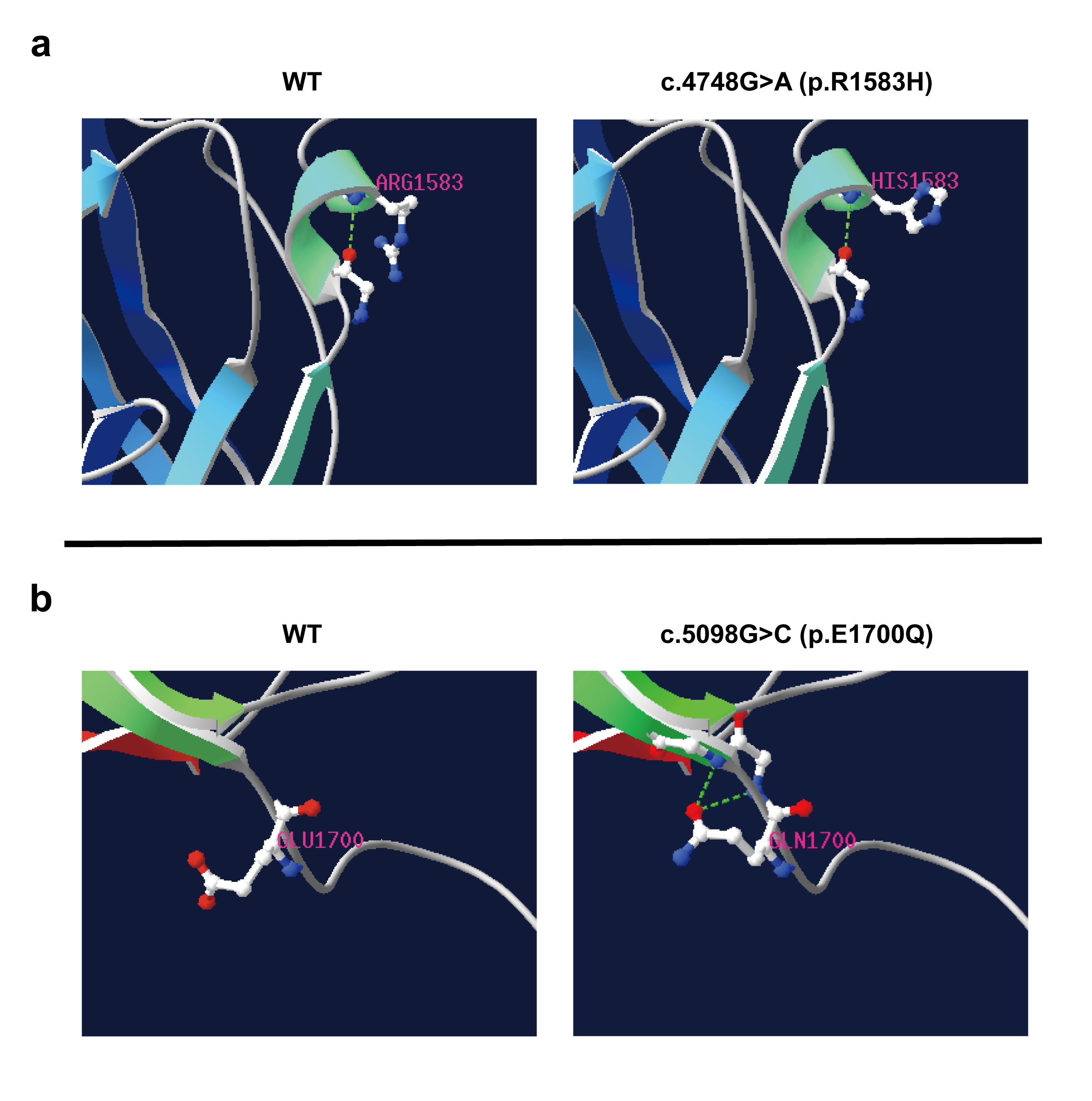

Supplement: Supplementary Materials — See Supplementary Figure 1 in the Supplementary Material. Local predicted protein tertiary structures of human otoferlin with p.R1583H and p.E1700Q. (a) Amino acid side chain of p.R1583H. Left: wild-type p.R1583. Right: mutation p.H1583. (b) Amino acid side chain of p.E1700Q. Left: wild-type p.E1700. Right: mutation p.Q1700. WT: wild-type. [file 9765276.f1.tif]
